# Systemic and local lipid adaptations underlie regeneration in *Drosophila melanogaster* and *Ambystoma mexicanum*

**DOI:** 10.1038/s41536-024-00375-x

**Published:** 2024-10-29

**Authors:** Ines C. Kübler, Jenny Kretzschmar, Maria Nieves Arredondo-Lasso, Sean D. Keeley, Luca Claudia Rößler, Katharina Ganss, Tatiana Sandoval-Guzmán, Marko Brankatschk

**Affiliations:** 1 Department of Internal Medicine III, University Hospital Carl Gustav Carus, Technische Universität Dresden, Dresden, Germany; 2 Biotechnology Center (BIOTEC), Technische Universität Dresden, Dresden, Germany; 3 MRC Laboratory of Molecular Biology, Francis Crick Ave, Trumpington, Cambridge, UK; 4 Metabolic Programming, Technische Universität München, Freising-Weihenstephan, Germany; 5 Paul Langerhans Institute Dresden, Helmholtz Centre Munich, University Hospital Carl Gustav Carus, Technische Universität Dresden, Dresden, Germany; 6 Center for Regenerative Therapies TU Dresden, Technische Universität Dresden, Dresden, Germany; 7 Faculty of Biology Technische Universität Dresden, Dresden, Germany. Zellescher Weg 23b, Dresden, Germany

**Keywords:** Fat metabolism, Regeneration

## Abstract

In regenerating tissues, synthesis and remodeling of membranes rely on lipid turnover and transport. Our study addresses lipid adaptations in intestinal regeneration of *Drosophila melanogaster* and limb regeneration of *Ambystoma mexicanum*. We found changes in lipid profiles at different locations: transport, storage organs and regenerating tissues. We demonstrate that attenuating insulin signaling, exclusively in fat storage, inhibits the regeneration-specific response in both the fat storage and the regenerating tissue in Drosophila. Furthermore, in uninjured axolotls we found sex-specific lipid profiles in both storage and circulation, while in regenerating animals these differences subside. The regenerating limb presents a unique sterol profile, albeit with no sex differences. We postulate that regeneration triggers a systemic response, where organs storing lipids play a significant role in the regulation of systemic lipid traffic. Second, that this response may be an active and well-regulated mechanism, as observed when homeostatic sex-differences disappear in regenerating salamanders.

## Introduction

During their lifetime, multicellular organisms experience tissue damage at different scales. To survive, animals react in two different ways to wounding - by scar formation or functional tissue repair by regeneration. Although regeneration is performed by the majority of eukaryotes to some extent, many organisms are limited in their potency and only recreate particular cellular barriers^1^.

However, species exist throughout major lineages of the eukaryotic tree of life that exhibit great regenerative abilities. These include protostomes, such as numerous types of planaria, cephalopods, and arthropods, as well as deuterostomes, like certain fish, salamanders, and starfish. This broad extent of strong regenerative potential across lineages has inspired many studies trying to examine which features may be conserved, including gene expression, tissue organization, or maintenance of specific cell types^2^. One major factor that has so far been often overlooked is whether major changes in systemic metabolic processes are able to support a regenerative response^3^. To drive cell proliferation and growth necessary to replace lost tissues, substantial production of cellular components - such as lipids and amino acids - is required. In addition, the presence of signaling cues inducing cellular differentiation and function is essential at the injury site. Regenerating tissues recruit macronutrients, such as glucose and lipids, from the circulatory system to fulfill changes in energy demands. To maintain the flow of nutrients, the regenerating organism is likely to adopt an altered metabolism compared to what is observed during homeostasis. We speculate that these changes are manifested in metabolic organs such as the liver and white adipose tissue.

Our study compares systemic lipid changes associated with regeneration, induced by chronic intestinal inflammation in fruit flies^4^ or by limb amputation in axolotls^5^. We parallel both experimental paradigms at a phase of high stem cell/progenitor proliferation, and analyze how the transport and storage of lipids correlate with lipid content at the injury site. Our results highlight the importance of sex-specific adaptations in lipid metabolism to facilitate functional tissue repair, as well as the necessity of AKT signaling and sterol homeostasis.

## Results

### Systemic transport of saturated fatty acids lessens in regenerating Drosophila

Intestinal cells are exposed to chemical and mechanical stress, and damaged cells are constantly replaced by proliferation of intestinal stem cells (ISC). One well studied model of intestinal regeneration is adult Drosophila flies fed with food supplemented with the drug dextran sodium sulfate (DSS)^6,7^. In Drosophila, feeding behavior is sex-specific, in brief, males feed at irregular intervals alternating with roaming behavior (Supplementary Movie 1), while mated females show a constant food intake and less mobility (Supplementary Movie 2). In this study, we use mated females to exclude metabolic differences created by the feeding behavior. Continuous intestinal tissue damage could influence feeding behavior and nutritional input, thus, we monitored the food intake of DSS-treated flies using the CApillary FEeder assay (CAFE)^8^ and found no changes in feeding behavior with respect to controls (Supplementary Fig. 1a). In addition, blood sugar and protein content of regenerating flies were comparable to fed wild type controls but noticeably higher than in starving wild type animals (Supplementary Fig. 1b, c), indicating that the function of the interstitial epithelium is not compromised by DSS administration. Further, DSS treatment of adult flies did not impede viability (Supplementary Fig. 1d). However, our assay induces a proliferative response, validating the use of this assay as a regenerative model (Supplementary Fig. 1e).

**Fig. 1:**
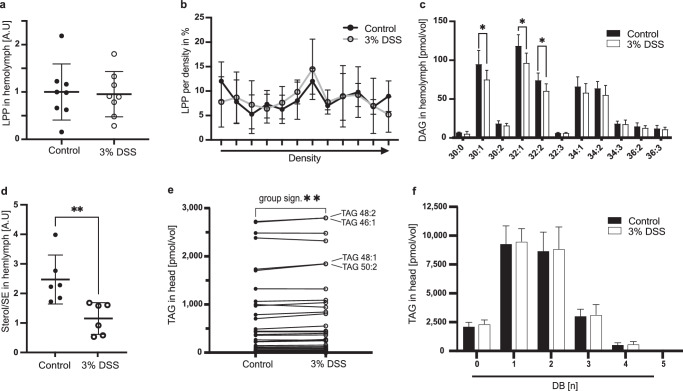
Systemic lipid transport in regenerating *Drosophila melanogaster.* **a** Total amount of Lipophorin (LPP) in Drosophila hemolymph in control and regenerating animals. (*n* = 8, 9 flies pooled per sample, 3% DSS=regeneration). Unpaired Student’s t-test. Mean and SD are indicated. **b** LPP particles present in hemolymph, separated in a density field. (*n* = 4, 27 flies pooled per sample). Arrow points to high density. Multiple unpaired Student’s t-tests. Mean and SD are indicated. **c** DAG yields in hemolymph (*n* = 6, 3 flies pooled per sample, 3% DSS=regeneration). Multiple unpaired t-tests with Welch correction, *p < 0.05. Mean and SD are indicated. **d** Circulating Sterols in hemolymph normalized to the amount of sterol esters (SE). *n* = 6, 5 flies pooled per sample, unpaired Student´s t-test, **p < 0.01, mean and SD. Mean and SD are indicated. **e** TAG yields in head samples of control and regenerating flies. Identical lipid species are connected using a line to visualize their individual change between the mean of the two experimental groups. Wilcoxon test for total TAG changes, p** < 0.01. *n* = 6, 3 heads pooled per sample. **f** Saturation profile of all measured TAG species from Drosophila head samples. DB double bonds. Mean and SD are indicated.

Although Drosophila flies are sterol auxotrophs, they are able to synthesize saturated and mono-unsaturated fatty acids^9^. We expected that our experimental flies feeding on lipid-free food (see material and methods) will be starved of sterols and poly-unsaturated fatty acids. Further, we speculated that with the loss of dietary lipid (DL) flows, we would easily detect if a systemic re-distribution of essential lipids in regenerating flies takes place. To investigate changes in circulatory lipid contents, we isolated blood, named hemolymph, of adult flies and probed for lipoprotein particles, the principal systemic lipid carriers. In fruit flies, the lipoprotein Lipophorin (LPP) is responsible for carrying the bulk of diacylglycerols (DAGs) and sterols to supplement target cells with lipids^10,11^. First, we measured the number of LPP particles and their lipid load. Quantification of total hemolymph LPP by western blotting showed no differences between regenerating and control flies (Fig. 1a). To investigate the LPP density distribution, we isolated hemolymph and separated LPP in density gradients and found no difference in the LPP density profile in regenerating flies with respect to untreated controls (Fig. 1b). To assess the lipid load of LPP particles we extracted lipids from our samples and quantified glycerophospholipids and DAGs using mass spectrometry (Supplementary Data 1). We found no changes in the glycerophospholipid distribution of lipoproteins (Supplementary Fig. 2e, lower panels). However, regenerating animals transport less DAGs compared to non-regenerating animals (Supplementary Fig. 2f), and recorded a significant decrease in three of the most prominent DAG species: DAG^C30:1^ (*n* = 6, regenerating = 75.38 pmol/vol, control = 95.25 pmol/vol, *p* = 0.049 unpaired t-test with Welch correction), DAG^C32:1^ (*n* = 6, regenerating = 96.99 pmol/vol, control = 119.0 pmol/vol, *p* = 0.019 unpaired t-test with Welch correction) and DAG^C32:2^ (*n* = 6, regenerating = 60.78 pmol/vol, control = 74.48 pmol/vol, *p* = 0.027 unpaired t-test with Welch correction) (Fig. 1c). These DAG species consist mostly of saturated and mono-unsaturated fatty acids. In contrast, we did not find a significant reduction of DAGs composed of poly-unsaturated fatty acids (PUFAs). Lastly, we measured by thin layer chromatography (TLC) the sterol and sterol ester content in hemolymph of regenerating flies. Sterols form the lipid shell of LPP particles, whereas sterol esters resemble cargo lipids. We detected a decrease in the sterol:sterol ester ratio in regenerating flies indicating that the proportion of transported sterol esters is increased (Fig. 1d). Thus, our results indicate that regenerating flies are either incapacitated in their synthesis of fatty acids or the use of saturated fatty acids is exceeding the mobilization capacity from lipid stores. Further, the proportional increase in sterol esters points to compensatory transport possibly required to maintain cellular sterol homeostasis.

### Fatty acid stores increase in fat body cells

**Fig. 2:**
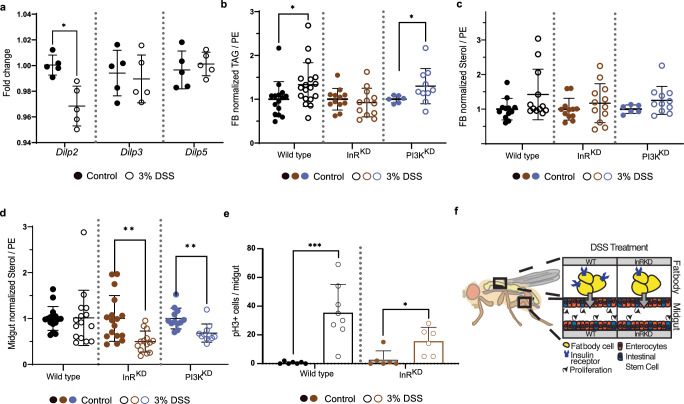
InR knock-down in the fat body modulates regeneration and lipid storage. **a** Expression of insulin-like peptides (Dilps) measured by qPCR. The expression in regenerating (3%DSS, circles) animals of *Dilp2*, *Dilp3* and *Dilp5* is shown as fold change to control (dots). (*n* = 5, each sample = 5 animals pooled, unpaired t-test, *p < 0.05). Mean and SD are indicated. **b** TAG yields normalized to PE and the average corresponding control signal in fat body samples of WT (black), FB-InR^KD^ (*lpp* > *InR*^*RNAi*^, brown) and FB-PI3K^KD^ (*lpp* > *PI3K*^*RNAi*^, purple) flies during control conditions (dots) and regeneration (3% DSS, circles). -Welch’s t-test, *p < 0.05). *n* = 15 (WT control), 18 (WT 3% DSS), 12 (InR^KD^, control and 3% DSS), 6 (PI3K^KD^ control) and 10 (PI3^KD^ 3% DSS), 3 animals pooled per sample. Mean and SD are indicated. **c** Sterol yields normalized to PE in fat body samples. Sample description, see (**b**). *n* = 15 (WT control), 18 (WT 3%DSS), 12 (InR^KD^, control and 3% DSS), 6 (PI3K^KD^ control) and 10 (PI3^KD^ 3% DSS), 3 animals pooled per sample. Mean and SD are indicated. **d** Sterol yields normalized to PE in midgut samples. Sample description, see (ba)., Welch’s t-test **p < 0.01. *n* = 16 (WT control), 15 (WT 3% DSS), 17 (InR^KD^, control), 14 (InR^KD^ 3% DSS), 12 (PI3K^KD^ control and 11 (PI3^KD^ 3% DSS). 2 animals were pooled per sample. Mean and SD are indicated. **e** Quantification of pH3 positive cells per midgut in WT and InR^KD^ flies in control condition and upon regeneration. Welch’s t-test, **p < 0.001, *p < 0.05. Wild type: *n* = 7 (Control), *n* = 8 (3% DSS); InR^KD^: *n* = 6 (both conditions). Mean and SD are indicated. **f** Graphic overview depicting the InR knock-down strategy in the fat body cells. DSS treatment in the InR^KD^ results in markedly reduced intestinal stem cell division rates in regenerating animals.

In mammals and insects, liver-like cells are the main provision stock of low-density lipoproteins (LDL)^11–13^, and intestinal cells or white adipocytes represent the source for the majority of circulating lipids^11,14^. In Drosophila, fat body cells (FB) form functional hybrids of vertebrate hepatocytes and white adipocytes. To investigate if the reduction of saturated DAGs in circulation is correlating with changes in respective fatty acids in FBs we measured the lipid content of these cells using mass spectrometry (Supplementary Data 1). We analyzed the lipid composition of head samples due to their distance from the regenerating intestine. The majority of triacylglycerols (TAGs) measured in head samples originate from FB cells. In head samples, we found that total TAG levels are significantly higher in regenerating flies (Figs. 1e and 2b, wild type panel). Interestingly, the increase in TAG was driven by TAG species likely composed of FAs with 14 or 16 carbons (Fig. 1e, TAG species indicated). However, regeneration did not induce a change in the general saturation profile of TAGs (Fig. 1f).

Our data rejects the idea that FA synthesis is reduced in regenerating flies. Although correlative, our results indicate that especially systemic traffic and hepatic storage saturated/mono-unsaturated medium-chained FAs are regulated by regeneration. Thus, we speculate that the reduction of circulating saturated DAGs (Fig. 1c) is likely based on increased storage of respective FAs in cells. Earlier, we have measured increased sterol ester levels in circulation (Fig. 1d). Unfortunately, the presence of wax in the tracheal cuticle does not permit a clean sterol ester measurement in Drosophila tissues. Nevertheless, we found sterol levels not affected in FB of regenerating flies (Fig. 2c, wild type panel). Taken together, we speculate that regenerating flies remove saturated and mono-unsaturated FAs selectively from the system by storing these lipids in FBs.

### Insulin signaling in fat body cells regulates lipid content in regenerating tissue

Insulin signaling is uniquely important for storing energy as fat in humans. In Drosophila, FB cells are reported to change lipid turnover and the storage of TAG in response to insulin^15^. To investigate if the expression of insulin-like peptides (Dilp) is affected by regeneration, we measured relative mRNA of *Dilp2*, 3 and 5 by qPCR. Our results show upregulation of *Dilp2* expression while *Dilp3*, and 5 remain unchanged (Fig. 2a). Insulin is required for ISC division in Drosophila^7^. We found that regenerating female flies increase specific TAGs in fat body cells (Fig. 2b) and less sterol esters are transported systemically (Fig. 1d). Insulin signaling is regulating TAG storage and thus, we speculated that impaired insulin signaling in FB cells could affect lipid homeostasis. We focused on the fact that regenerating animals decrease systemic transport of saturated FAs and increase sterol traffic. One rationale for modifying lipid transport may arise from the specific needs of regenerating tissues at the local level. To test the idea, we knocked down the insulin receptor specifically in FB cells (FB-InR^KD^). We found that sterol levels are not affected in the FB of intact or regenerating flies (Fig. 2c, mid panel). However, when we measured sterol levels in intestinal cells, we found a profound decrease in sterols in midguts from regenerating FB-InR^KD^ flies (Fig. 2d mid panel). To confirm the result, we impaired the expression of a downstream factor of the insulin signaling cascade in fat body cells, the phosphoinositide 3-kinase (PI3K). We found that FB-PI3K^KD^ cells mimic intestinal sterol changes found in FB-InR^KD^ (Fig. 2d right panel). This suggests that insulin signaling in the FB, required to maintain intestinal sterol homeostasis, is regulated by the metabolic branch of the insulin signaling pathway. To investigate if preventing the systemic regeneration-driven lipid adaptations affects intestinal regeneration, we assessed the ISC division rate in midguts. We found that ISC in regenerating FB-InR^KD^ flies decreased their proliferation rate by about 50% compared to the wild type condition (Fig. 2e). While DSS treatment in wild type flies induced an increase in proliferation (Fig. 2e left panel), this increase in proliferation was significantly reduced in the FB-InR^KD^ condition (Fig. 2e right panel). Taken together, induced intestinal regeneration requires insulin signaling in FB cells to regulate sterol dynamics of midgut cells (Fig. 2f). However, we cannot rule out that changes in insulin signaling may affect cell numbers in the FB, thus reflecting different lipid content. Therefore, we normalized our sterol readings to membrane lipids (PE). Adaptations of sterol metabolism should not be surprising, since cellular sterol levels are critical to allow cell functionality or proliferation^16^. Nevertheless, *Drosophila* as a sterol auxotroph, could potentially reflect insect-specific lipid managements in response to continuous regeneration. Further work is needed to quantify the regenerative success in intestines upon impaired insulin signaling in FBs.

### Regenerating axolotl show normal feeding behavior and nutrition

To test the translational potential of our findings in fruit flies, we studied limb regeneration in axolotls. The process of regeneration after limb amputation poses important differences from the Drosophila intestinal injury model, such as the complexity and multi-tissue content of the structure. In mice, findings indicate sex specific systemic lipid transport and lipid turnover^17^, therefore, we included both sexes in our study. We used axolotl larvae, 6 days after injury (dpa) when a transitional structure begins to form, the blastema, containing an initial rush of progenitor cells that will give rise to the new limb. During earlier timepoints, the main events are characterized by cell death and the initial immune response, with less proliferation. At later time points, a major remodeling event results in skeletal resorption and the beginning of differentiation^5^. For this study, we considered that the high proliferation rates at 6 days within the blastema and adjacent tissue in the stump, reflects the cell activation in response to the injury that we report in Drosophila and offers a point of interspecies comparison. Furthermore, at 6 dpa the expression of regeneration-specific markers can already be observed^18^. At this developmental stage, axolotls are fed with freshly hatched *Artemia franciscana* nauplii, rich in PUFAs and cholesterol (see materials and methods) - representing a homogenous, lipid-rich food (Supplementary Data 2). Thus, unlike Drosophila on lipid-free food, regenerating axolotls can rely on dietary lipid (DL) flows to control their lipid homeostasis. First, we controlled for the feeding behavior and nutrient content of regenerating axolotls. We videotaped foraging regenerating (5 dpa) and intact animals, *n* = 3. After 1 h, both experimental cohorts ingested the same amount of artemia as observed in these representative videos (Supplementary Movie 3−6, Supplementary Movie 3 = Male intact, Supplementary Movie 4=Male 5 dpa, Supplementary Movie 5=Female intact, Supplementary Movie 6=Female 5 dpa). Next, we measured circulating sugars and proteins. Similar to our findings in fruit flies, regeneration in axolotl larvae does not result in changes in blood sugar or protein levels (Supplementary Fig. 1f, g). In addition, we tested if regenerating axolotls experienced starvation by quantifying the expression of the transcription factor Forkhead box protein O1 (*Foxo1*) in the liver, which is known to increase its own expression profile in starving cells^19^. We did not detect significant mRNA changes in regenerating animals, indicating that limb amputation and subsequent recovery time did not induce malnutrition of experimental axolotls (Supplementary Fig. 3d).

### Systemic lipid transport is adjusted in a sex-specific manner

Regenerating tissues rely on high cellular proliferation rates and thus, we speculated that the flow of DLs are suitable resources to build cellular membranes. We investigated if lipid transport and compositions were different in 6 dpa animals with respect to intact axolotls. The molecular organization of systemic lipid transport in axolotls is largely uncharted. Therefore, we searched within the recently sequenced axolotl genome^20^ to discover which proteins axolotls encode that resemble lipoproteins of other species. We used reported protein sequences of identified lipoproteins from mice, frogs, and humans as templates and found homologous genes in axolotls, including *ApoB* and others (Supplementary Data 3). Thus, axolotls possess a more complex lipid transport than fruit flies and produce low- and high-density lipoprotein particles like other studied vertebrates^21^. Mass spectrometry showed that axolotl lipoproteins, similar to mammals, mainly transport TAGs, ~47 mol% (Supplementary Data 4).

**Fig. 3:**
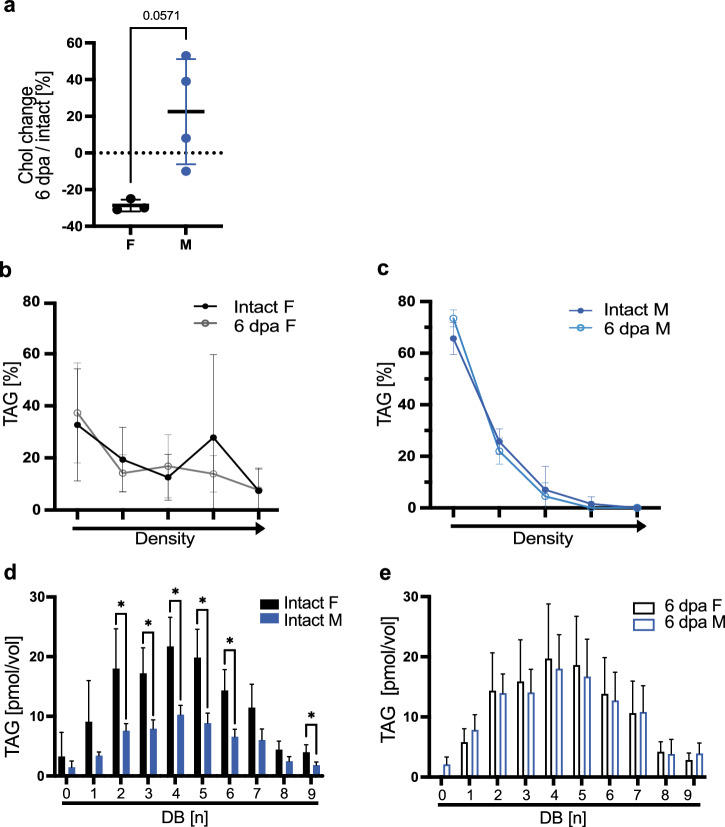
Systemic lipid transport in *Ambystoma mexicanum.* **a** Cholesterol content measured in axolotl plasma. Plotted as a percentage of the deviation from wild type cholesterol content (indicated as zero). Mean and SD are indicated. *n* female = 3 pools, from a total of 14 intact females or 14 6dpi females; male *n* = 4 pools from a total of 18 intact males or 18 6dpi males. Pools were created from animals of the same batch. Mann−Whitney test with *p < 0.05 or indicated. **b**, **c** TAG density distribution of female (**a**) and male (**b**) of axolotl plasma plotted as percentages of total TAG yields. Density gradient indicated by the arrow in the x axis. *n* = 6 per group. Mean and SD are indicated. Female (F), male (M). **d**, **e** Saturation profile of TAGs in axolotl plasma. *n* = 5, (**d**) comparison of intact F and intact M. **e** comparison of 6 dpa F and 6 dpa M. Statistical significance for each unsaturation group was determined via unpaired t-test with *p < 0.05 or indicated. Mean and SD are indicated. Female (F), male (M).

Given the more complex lipid transport, we additionally investigated if sex-specific differences in lipid transport exist, based on mice data showing sex-specific lipid turnover^17^. We speculated that adaptive metabolic changes equalizing sex specific turnover help synchronize tissue repair. Here, we demonstrate by TLC that intact and regenerating male and female axolotls predominantly transport sterol esters and sterols in LDL particles (Supplementary Fig. 2a−d). In addition, we determined plasma cholesterol levels using a colorimetric approach (Fujifilm DRI CHEM NX600V), and detected a consistent 30% reduced cholesterol in pooled samples of regenerating females with respect to intact siblings, whereas males show a slight increase (Fig. 3a). Males rely exclusively on LDL particles to traffic TAGs systemically (Fig. 3c). These lipoprotein density profiles in males did not change during regeneration, suggesting a consistent approach to TAG transport that is independent of the injury status. In contrast, in female axolotls at homeostatic conditions and during regeneration, TAG transporting lipoproteins are spread across a wide range of densities, indicating they are transported by multiple different lipoprotein classes (Fig. 3b). In addition, mass spectrometry revealed that the content of trafficked TAGs in axolotls is also sex-specific: Female axolotls have more TAGs in their circulatory system than males and in consequence, traffic more polyunsaturated fatty acids (PUFAs)(Fig. 3d). Following amputation, regenerating female axolotls do not change systemic TAG transport and show TAG profiles similar to non-regenerating controls. Astonishingly, the difference between females and males in intact conditions, recedes during regeneration as males increase circulating TAG yields and the proportion of PUFAs (Fig. 3e). This increase aligns male TAG levels with those of females, potentially indicating that such levels are optimal for regeneration. In both sexes, the majority of TAG species belong to TAG^C52^ and TAG^C54^, which represent about 78% of all plasma TAGs (Supplementary Data 4, Supplementary Fig. 2g). Further, we detected the glycerophospholipid phosphatidylserine (PS) in the plasma of intact females but not in males. Interestingly, following amputation, PS were not found in regenerating female samples, but did appear in circulation of regenerating males (Supplementary Fig. 2e top panel). As PS are found primarily on high density particles (HDLs)^22^, these findings further underline the existing substantial sex-specific differences in lipid transport. In contrast to TAGs and PS, sterol and SE levels in circulation remain unchanged between all experimental axolotl groups (Supplementary Fig. 2h).

We show that in homeostasis, axolotls possess a systemic sex-specific, lipoprotein-based, lipid transport, and we conclude that systemic traffic of fatty acids and sterols are likely not directly linked. Further, lipid transport is also dynamically different in regenerating males and females however, equaling the initial homeostatic difference.

### Liver serves as systemic sterol buffer in females

**Fig. 4:**
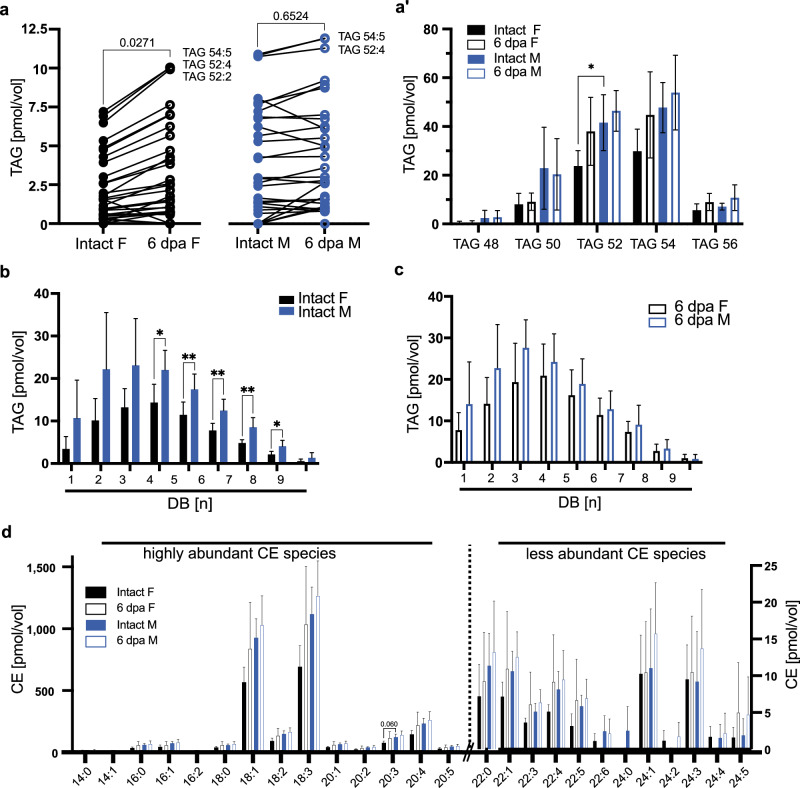
Regeneration equalizes sex differences in lipid storage in the axolotl liver. **a** TAG yields in liver samples of control and regenerating female and male axolotls. Same lipid species are connected with a line to visualize their individual change between the two experimental groups. Most abundant lipid species are labeled. *n* = 6. Student’s t-test to compare between intact F and 6 dpa F and between intact M and 6 dpa M. **a**’ Data in (**a**) graphed by TAG species including all groups of DB. *n* = 6, per species one-way ANOVA and Tukey´s multiple comparison test. *p ≤ 0.05. Mean and SD are shown. Female (F), male (M). **b**, **c** Quantification of stored lipids in the axolotl liver by the degree of unsaturation via mass spectrometry. Comparison between (**b**) intact male and intact female, (**c**) regenerating male and female animals. *n* = 6 per group. Statistical significance for each unsaturation group was determined via unpaired t-test. For all graphs, the mean and SD are shown. **d** Cholesteryl ester species yield from female and male axolotl samples (*n* = 6). Kruskal-Wallis and Dunn’s multiple comparison test, *p < 0.05 or indicated. SD and mean are indicated. Female (F), male (M).

To assess if changes in lipid transport exert influence in lipid storage, we first established where fat is stored in axolotl larvae. Histological analysis shows that at this developmental stage, white adipose tissue is largely scarce^23^ (and own observations). However, we found that liver cells are strongly enriched with TAGs and sterol esters (Supplementary Fig. 3a, b), concentrating lipid storage in one organ, thus facilitating a parallel to FB cells in fruit flies. The hepatic lipid profile of intact animals showed that female livers contain lower amounts of unsaturated TAGs, compared to males (Fig. 4a) (Supplementary Data 5) and that one of the most abundant species, TAG^C52^, is significantly different between females and males (Fig. 4a′). However, limb regeneration in females induced an increase in TAG and PUFAs with respect to controls (Fig. 4b, c). In addition, we found an increase in hepatic cholesteryl esters (CEs) of females, while they remain unchanged in males, resulting in a similar profile for high and low abundant CEs in both sexes during regeneration (Fig. 4d). CEs represent a biological storage form of sterols and on demand, CEs can be converted into cholesterol and free FA. An increase in CEs could point to raising cholesterol levels, however, there is the possibility that the axolotl sterol profile is not dominated by cholesterol^24^. Thus, we used TLC to assess total sterol levels in the axolotl liver, which showed that sterol yields of female or male hepatocytes are not increasing in regenerating animals (Supplementary Figure 3c). Hence, we suggest that regenerating axolotls are capable of buffering increasing hepatic cholesterol levels by converting super-numerical sterol molecules into sterol esters.

### AKT-dependent signaling is increased in the liver of regenerating females

We speculated that insulin signaling is regulating lipid turnover in axolotl hepatocytes analogous to our findings in FB cells of fruit flies. Thus, we predicted, based on observed lipid changes, to find profound adjustments of the insulin signaling cascade between regenerating females and males. To test our idea, we first identified the kinases AKT and annotated MAPK in the axolotl genome (Supplementary Data 6). AKT regulates the metabolic branch of the insulin cascade, whereas MAPK regulates the mitogenic pathway. To compare intact to regenerating animals we loaded equal amounts of protein (1 mg/ml) on a Western blot, and confirmed loading with a corresponding protein stain (Supplementary Fig. 4a’, b’). We show that intact female axolotls express a low baseline of AKT protein when compared to male siblings (Supplementary Fig. 4a). However, in regenerating females we observed a dramatic increase of AKT levels. Conversely, MAPK is expressed in females and males, but MAPK protein levels remain unchanged in 6 dpa hepatocytes with respect to intact animals (Supplementary Fig. 4b). This protein detection is not specific to the activated form of the proteins, therefore, we proceeded to investigate a downstream effector, *Foxo1*. Our data shows that in intact female and male axolotls, *Foxo1* expression correlates to the tendency of higher AKT protein content in males. However, we did not detect significant mRNA changes in the injured group, indicating that during regeneration, *Foxo1* expression does not report AKT activity or alternatively, AKT targets other effectors^25^ than FOXO (Supplementary Fig. 3d).

### State of sterol content in limb blastema is regeneration specific

**Fig. 5:**
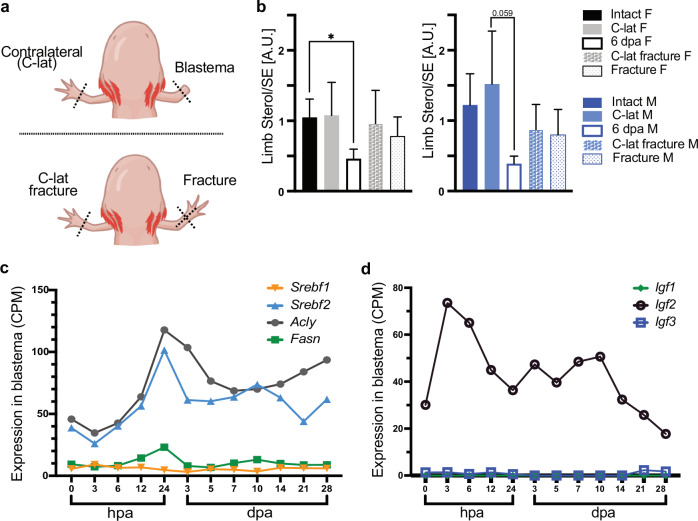
Sterol metabolism in limb blastema. **a** Graphical depiction of the experimental set-up for (**b**). **b** Sterol/SE ratio in intact limb and regenerating blastema, or in limbs with a fracture. A.U. arbitrary unit. *n* = 8, Kruskal-Wallis and Dunn´s multiple comparison test, *p ≤ 0.05. SD and mean are indicated. Female (F), male (M). **c** Expression of *Srebf1* and 2, *Acly* and *Fasn* in limb blastema during regeneration. Data obtained from Stewart et al.^26^. Counts per million (CPM) transcripts, hours post amputation (hpa), days post amputation (dpa). **d** Expression of *Igf*1-3 in limb blastema during regeneration. Data obtained from Stewart et al.^26^. Counts per million (CPM) transcripts, hours post amputation (hpa), days post amputation (dpa).

In the growing blastema, we found a significantly lower sterol: sterol ester ratio (driven by the increase of sterol esters) with respect to undamaged tissue. This sterol ratio is not replicated if animals had only a skeletal injury (fracture) instead of an amputation (Fig. 5a, b). To investigate if sterols are mobilized from tissues with high sterol content, we sampled dorsal and ventral muscle wall from regenerating animals and found no significant differences between groups (Supplementary Fig. 4c). Using existing data sets^26^, we investigated if lipid and sterol metabolism enzymes are correspondingly modified at the injury site. We found that sterol regulatory element-binding factor 2 (*Srebf2*) is upregulated at 24 h after amputation, and after a moderate down regulation, it remains above control levels up to 28 post amputation (Fig. 5c). This trend is mimicked by a target of SREBF2, the ATP citrate lyase (*Acly*), a fatty acid biosynthetic enzyme. Fatty acid synthase (*Fasn*), doubles its expression by 24 h after amputation, and at 3 dpa returns to control levels. In contrast, *Srebf1* is also detected in the blastema, but remains unchanged during regeneration. Since *Srebf* expression can be induced by insulin growth factors (IGFs)^27–29^, we investigated if IGFs would change during the course of regeneration locally in the injury site. Using existing data sets^26^, we found that only *Igf-2* is upregulated at the injury side in the hours prior to the *Srebf* peak (Fig. 5d). Taken together, we propose that axolotl blastema cells require a specific sterol homeostasis to drive efficient proliferation and differentiation. Further, we postulate that the sterol homeostasis in blastema cells is aided by systemic sterol transport regulated by hepatocytes, however, there is an activation of the fatty acid-based synthesis machinery at the injury site.

## Discussion

Functional tissue repair requires active and controlled cell proliferation and differentiation of one or several cell lineages. This implies readily available biomolecules to ensure duplication of DNA, formation of new membranes, deposition of supporting extracellular matrix, and protein production on demand. In consequence, the affected organism needs to be capable of providing the sufficient energy and building blocks. In this work, we show that regenerating mated Drosophila females and axolotls use lipid metabolism to flexibly adapt to the energetic demand of high cell proliferation. Both organisms continue to feed and absorb food ensuring a continuous influx of macronutrients. Further, we demonstrate that blood sugar and protein levels remain comparable to wild type/uninjured animals, indicating that these nutrients are consistently available in regenerating tissues. However, regenerating animals could have a different protein profile in circulation, which was not assessed in this work. In contrast, regenerating fruit flies and axolotl larvae adapt systemic lipid transport and hepatic lipid storage. We also show that insulin signaling in hepatic-like cells is an important factor to maintain a flexible, systemic lipid metabolism, and preventing these adaptations disrupts proliferation in regenerating midguts. Finally, our study demonstrates a homeostatic sex-specific difference of the lipid metabolism that subsides in regeneration. Overall, our study demonstrates that, remarkably, two species as different as flies and axolotls, in response to a different injury and fed with diverse diets, consequently respond to injury with dynamic systemic lipid adjustments, favoring a sustained sterol metabolism in the injured tissue. It remains to be shown if regeneration in other tissues would exert the same systemic and local lipid adaptations.

While both species modify their lipid storage and circulation, changes at the site of injury are only observed in axolotls. This could be explained by the different amount of biomass in a relatively short time that regenerating a limb requires. In Drosophila, regenerating intestines show no sterol difference to intact guts. However, impairing insulin signaling in FB cells of regenerating animals results in intestinal sterol accumulations and decreased cell proliferation. Considering that regenerating wild type flies transport more sterol esters systemically and that fruit flies do not synthesize sterols, our results strongly indicate that regeneration actively utilizes sterol metabolism, mirroring our findings in limb regeneration in axolotl. In other words, likely a needed quota of sterols:sterol esters is needed at the injury site and storage organs actively mobilize lipids to this end.

We charted lipid transport and storage in axolotl, and this allowed us to study lipid metabolism changes in response to regeneration targeting only one specific organ. In Drosophila, FB cells are functional analogues of hepatocytes and white adipose tissue. Notably, we show that axolotl larvae rely on liver cells as main fat storage. Although liver as the main fat storage has been reported in sharks^30^ and frog tadpoles^31^, it was unknown if liver lipid storage would allow the axolotl to mobilize lipids on demand. We show the buildup of TAG in hepatic storage organs of regenerating animals. The hepatic accumulation of TAGs can be explained by increased FA delivery, hepatic FA synthesis or reduced TAG mobilization^32^. In flies, we measured a significant decrease of specific lipid species composed of medium-chain saturated fatty acids and a correlating increase in respective TAGs in FA cells. Thus, we conclude that FB cells capture saturated FAs from circulation, immobilizing these lipids.

The buildup of cellular TAG stores is regulated by AKT, central to the metabolic signaling branch downstream of the insulin receptor^33^. We found that hepatocytes of regenerating female axolotls increase total AKT levels. With this increase, hepatocytes of regenerating females reach AKT levels found in regenerating male animals. AKT detection per se is not indicative of increased AKT activity, since AKT function is regulated by reversible phosphorylation at dedicated amino acid positions. Thus, it will be important in the future to develop the tools to detect these post-translational modifications in the axolotl. We found that regenerating female flies upregulate *Dilp2* expression, and the FB specific knock-down of the insulin receptor or PI3K impairs sterol homeostasis in intestinal cells. PI3K is a factor of the cascade downstream of the insulin receptor and converts the membrane lipid Phosphatidylinositol 4,5-bisphosphate into Phosphatidylinositol (3,4,5)-trisphosphate required to recruit and activate AKT. AKT mediated signaling represents the metabolic branch of insulin signaling known to promote the formation of storage lipids.

One open question is, how an injury would influence insulin-like signaling required to re-organize systemic lipid turnover. Injury opens an invasion route for microorganisms and initiates an immediate immune-response. In Drosophila, it has been shown that the inflammatory *immune deficiency* pathway (IMD) increases *Dilp2* expression in insulin producing cells^34^. In insects, some bacteria stimulate intestinal lipid production and induce lipid accumulation in FB cells^35^, and in turn, insulin signaling regulates FBs promoting a mounted immune response to bacterial infections^36,37^. Supporting this insulin-immune response loop, intestinal damage and contact with gram negative bacteria stimulate the IMD-pathway and change insulin signaling^38^. Interestingly, we found that the regenerating limb in axolotl increases the expression of *Igf-2*, and while we did not identify the cell source, it has been shown that muscle cells, fibroblasts or macrophages can represent a rich source of IGFs^39,40^. This is relevant because IGF-2 in circulation can modulate growth^41^ and is able to bind with high affinity to insulin receptor isoforms^42^, and activate insulin signaling in hepatocytes^43^. Thus, if IGF-2 produced locally, enters circulation it could turn into a systemic signal modulating liver function, a possibility that should be further explored. Taken together, we speculate that the interaction with microbes at the injury site results in the activation of the immune system that results in metabolic changes including modulations of the systemic insulin signaling.

Multiple studies have demonstrated sex-dependent differences in wound healing^44^, regeneration^45,46^ or stem cell proliferation potential^47,48^. However, the underlying mechanisms resulting in this sex-dimorphism are often poorly understood and there is growing evidence that it cannot exclusively be explained by direct steroid hormone effects^45,49,50^. It is worth noting that axolotls in this study are still in the early growth phase and far from reaching sexual maturation, yet, the sex differences are prominent. We show that in axolotl there is a homeostatic sex-difference, in storage and circulation, and this difference equalizes in response to regeneration. Females masculinize their storage lipid profile, while males feminize their circulating lipid profiles, suggesting an active and regulated mechanism and potentially indicating an optimal condition for regeneration. It remains to be explored if the similar growth rate of the limb in both sexes that we have observed stems from the metabolic plasticity of female axolotls.

Another observation of interest is the switch in sterol and PUFA traffic in regenerating tissues and lipid storage organs is potentially similar to findings in tumor cells^51^. Cellular lipid synthesis in proliferating cells is regulated by several cues. Sterol regulatory element binding proteins (SREBFs), which are transcription factors responding to cellular sterol and PUFA content. Activated SREBF signaling results in increased biosynthetic rates promoting cell survival and division^1,51,52^. In support, we demonstrated the essential requirement of sterol homeostasis in regenerating intestinal cells of Drosophila. In the axolotl genome, we identified *Srebf1* and *2*, and their proposed transcriptional targets *Acly* and *Fasn*. Analyzing transcriptional data from limb blastema at different regeneration stages, we found specific upregulation of *Srebf2* and components of the FA synthesis machinery after injury. Thus, we propose that hepatic cells participate in the sterol-homeostasis of regenerating tissues, required to foster the production of phosphoglycerides (as shown by the upregulation of *Fasn* in blastema cells, Fig. 5c), which are essential to build membranes. Furthermore, in axolotl, this regulation is sex-specific to support a sex-independent regeneration lipid profile.

Our approach allowed us to parallel regeneration of an internal organ and an external structure, we based our comparison on a high proliferative phase of regeneration while monitoring for food intake and starvation signs. Additionally, our models concise the analogous physiological function of liver and adipose tissue in one organ, facilitating our analyses. However, our comparison limits our ability to answer some generic questions. Foremost, dietary lipids are one recognizable source to deliver energy, to form structural building blocks for cellular membranes or to function as signaling cues^16^. Although we have kept regenerating Drosophila on lipid-free food, we are unable to exclude the possibility that some dietary lipids can influence regeneration. Our study focused on the initial response during regeneration, more studies should address how the organism brings back metabolic homeostasis in the sustained growth phase of limb regeneration. Additionally, the use of axolotl larvae could limit us from making general statements of lipid adaptation during regeneration in sexually mature animals. Nevertheless, our two animal models, at different developmental stages, demonstrate that regeneration shows a remarkably conserved plasticity in lipid metabolism.

## Methods

### Animal models and husbandry

Flies (*Drosophila melanogaster*) were raised and bred on normal food^48^ at 25 °C maintaining a regular day/night cycle until used for experimental procedures. 12 (9 female and 3 male) 5- to 10-day (d) old flies were transferred to 1% agar plates. To tightly control the dietary input, protein-sugar-rich food (60 g/l soy peptone (Carl Roth), 90 g/l sucrose (Kaufland Classics), 582 kcal/l) was provided as liquid in wells, containing 3% DSS^7^ (MW: 35−50 kDa, MP biomedicals) for inducing regeneration of the intestine in adult flies. Drug treatment and control experiments were performed at 20 °C for 4 days. Unless specified otherwise, mCherry:foxo flies were used for all experiments (#80565, Bloomington). Other fly lines used in this study: UAS:InR RNAi (#992, VDRC), LPP Gal4^10^. To control for genetic variations, flies were crossed back to *white*^*1118*^ for five generations before experiments commenced. To evaluate the food uptake of flies, we performed the CApillary FEeder assay (CAFE)^8^. In short, 12 flies (9 females, 3 males) were placed in sealed vials and protein-sugar-rich food was provided in 5 μl microcapillaries. Flies were kept at 20 °C and capillaries were replaced every 24 h. The total food uptake was evaluated by comparing the reduction in food compared to an evaporation control. Further analysis was performed only in females.

Larval axolotls (*Ambystoma mexicanum*) were maintained at the axolotl facility at CRTD TU Dresden^53^. All procedures were performed according to the Animal Ethics Committee of the State of Saxony, Germany. We performed experiments using white axolotls (d/d) which were fed daily with freshly-hatched *Artemia franciscana* nauplii. For our experiments, we used larval axolotls of ~4.5 cm total body length. All samples were collected at the same time of the day and prior to their daily feeding. Axolotls were euthanized by exposing them to lethal anesthesia (0.1% benzocaine) for at least 20 min.

*Artemia franciscana* eggs, called cysts (Sanders Great Salt Lake Artemia Cysts), were cultured in 28 °C salt water for 48 h to allow hatching of the nauplii. Afterwards, they were harvested, transferred to 40% Holtfreter’s solution and fed to larval axolotls. The nutrient profile (total protein, sugar and lipid levels) of the artemia cyst have been determined by the manufacturer^54^. Animals were randomly allocated to experimental and treatment groups. Investigators were not blinded to allocation.

**Composition of artemia cysts**

**Table:** 

protein	>50.0%
fat	>19.0%
fiber	<2.0%
ash	>6.1%
moisture	<8.0%

**Lipid composition of artemia cysts**

**Table:** 

FA species	common name	mg/g
C18:0	stearic acid	9.0
C18:1	oleic acid	27.2
C18:3	linoleic acid	9.1
C18:4	stearidonic acid	11.2
C20:0	arachidic acid	1.1
C20:3	eicosatrienoic acid	2.7
C20:4	arachidonic acid	4.5
C20:4	eicosapentaenoic acid (EPA)	4.1
C22:6	docosahexaenoic acid (HDA)	0.1

### Live recording of feeding behaviors

To record feeding of *Drosophila melanogaster*, Petri-dishes were filled with 0.8% agar and a parafilm snippet was placed in the center. Onto the parafilm 10 µl liquid food bait was pipetted. Flies in a male:female ratio of 3:9 were video-recorded (1 frame/sec) for 2 h at 20 °C. Alternatively, each well of 24 well plate was filled with 0.8% agar, a food bait was placed in the center of each well and after 1 h incubation, the feeding behavior of single flies was recorded for 2 h at 20 °C. To record feeding of *Ambystoma mexicanum*, three-month old axolotl larvae were placed into containers and incubated for 30 min. Thereafter, the behavior of these animals was recorded initially for 15 min (1 frame/sec). Following the initial observation period, 1 ml of artemia-suspension was added and the feeding behavior was recorded for another 2 h.

### Assessment division rate intestinal stem cells *D. melanogaster*

Guts of adult, female flies were dissected in 4% paraformaldehyde in Grace’s insect medium (Lonza, #04-649 F) and incubated in fresh fix solution for 20 min at room temperature. After blocking with 5% native goat serum (NGS) in 0.1% Triton X-100/PBS overnight at 4 °C, incubation of primary and secondary antibodies was performed overnight at 4 °C. Antibodies were diluted in 5% blocking solution (5% NGS in 0.1% Triton X-100/PBS). Phospho-Histone H3 (Cell Signaling, #9706S, 1:500) was used as the primary antibody. Used secondary antibodies and fluorescent markers included: Alexa Fluor 488 (Invitrogen, #SG255489, 1:500) and DAPI (Sigma Aldrich, #28718-90-3, 1:1.000). Fixed guts were mounted in 50% Glycerol/1x PBS on microscope slides. Guts were imaged with Zeiss ApoTome1 (Carl Zeiss Microscopy) using the 20x/0.8 Plan-Apochromat air objective (Carl Zeiss Microscopy). The microscope was operated by ZEN blue (V2012 1.1.2.0) software. The number of phospho-Histone H3 positive cells was assessed manually in the whole midgut.

### Sample collection

To collect samples from flies, females were anesthetized using CO_2_. For head and hemolymph collection, flies were snap-frozen in liquid nitrogen. Fly heads were separated from bodies using forceps in liquid nitrogen. Heads and bodies were stored separately. Hemolymph was extracted from fly bodies by freeze-thawing on ice in 100 μl PBS, resulting in minimally TAG-contaminated samples. As TAG are stored in lipid droplets, a TAG-free sample indicates a low amount of cellular contamination. Hemolymph of 5 females pooled into 1 sample for sterol measurement by TLC, 9 female flies for protein measurements and of 27 flies for trehalose measurement was pooled per sample. Guts of adult, female flies were dissected in ice-cold Grace’s insect medium (Lonza, #04-649 F) (GIM). Two guts were pooled per sample. Fat body cells were collected by cutting through the flies’ cuticle and loosening fat body cells into 500 µl GIM. Five flies were dissected per sample, and 400 µl of FB cells containing GIM were taken per sample for further processing. All samples were stored at −80 °C until used for further experiments. To collect samples from *Ambystoma mexicanum*, blood was drawn directly from the heart with a heparin-coated 10 µl Eppendorf pipette tip and transferred to a heparin-coated Eppendorf tube. The blood was centrifuged at 4 °C 3.000 rpm for 10 min to separate plasma from blood cells. The supernatant plasma was transferred to a new heparin-coated Eppendorf tube. The plasma was either directly used for measurements or stored at −80 °C until usage. The axolotl liver, the blastema and the entire contralateral limb tissue were collected and stored in Eppendorf tubes at -80°C until usage.

### Ultracentrifugation of plasma and hemolymph

Hemolymph of 27 snap-frozen female flies was collected by freeze thawing on ice in 120 µl PBS. 100 µl of the hemolymph-PBS mix was used for ultracentrifugation. 5 µl axolotl plasma was diluted (5 + 95). Plasma and hemolymph samples were mixed with 3.5 ml salt mix (1.44 ml KBr 5 g/ml and 2.16 ml 1.6x PBS) in centrifugation tubes (Beckman Coulter 13 × 51 mm). The samples were separated by density via ultracentrifugation (Optima MAX-XP Ultracentrifugation Beckman Coulter with MLS-50 44.0 rotor; 268.000 × *g*, vacuum ON, deceleration ≤ 5) for ~16 h. Afterwards, the samples were split from top to bottom into fractions of 300 µl and stored in 2 ml Eppendorf tubes at −80 °C until usage.

### Quantification of circulating sugars (trehalose and glucose)

Quantification of trehalose levels in *D. melanogaster* was performed following the manufacturer protocol using a trehalose kit (Megazyme) following the manufacturer’s protocol. Hemolymph of 27 flies was pooled per sample.

Plasma glucose levels in *Ambystoma mexicanum* were determined enzymatically with a commercially available blood/plasma glucose detection device i.e., Accu Chek Aviva (Roche) and Contour (Bayer). 1 µl of plasma was used for the measurement.

### Quantification of total protein amounts in circulation and liver

For flies, the hemolymph of 9 female flies was extracted in 100 µl PBS by freeze-thawing on ice, followed by a chloroform/methanol protein extraction. The protein pellet was resolved in 80 µl 0.1% PBS Triton. Axolotl plasma was diluted 1:320 in 0.1% PBS Triton. Axolotl liver was homogenized in 30 µl 0.1% PBS Triton and 1 µl of the homogenate dissolved in 24 µl 0.1% PBS Triton was used for protein quantification. Quantification of the total protein amount was done using the Pierce™ BCA Protein Assay Kit (Thermo Fisher Scientific, cat. no. 23227). The 96-well plate was shaken shortly and then incubated for 30 min at 37 °C. The amount of protein was detected using the Tecan plate reader with the following parameters: 30 s shaking, 562 nm absorbance, 10 flashes. Measurements were performed as technical triplicates, and the average protein concentration was calculated relative to a BSA standard curve.

### Quantification of cholesterol in circulation

Axolotl plasma cholesterol levels were determined colorimetrically with the Fujifilm DRI CHEM NX600V device. Measurements were performed in 10 µl of plasma, therefore, plasma from batch siblings from the same experimental condition were pooled. The difference of regenerating and intact animals from each batch was calculated as a percentage to allow comparison between batches.

### Mass Spectrometry sample preparation

Three fly heads were pooled per sample and sent for mass spectrometry. Samples were dissolved in 300 μl PBS and 150 μl were used for analysis.

The axolotl liver was dissected, separated into two equal halves and stored at −80 °C until analysis. One half was used for mass spectrometry. Sample concentration: 5−10 mg/ml; volume used for analysis: 50 µl. For the plasma lipid analysis, 2 µl plasma was 1:20 diluted in PBS and used for the lipid analysis.

50 µl concentrated freshly-hatched artemia nauplii were homogenized and stored at −80 °C. Samples were diluted 1:6 and 75 µl were used for the analysis.

### Mass spectrometry measurements

Mass spectrometry was performed by Lipotype GmbH. Samples were stored at −80 °C. Prior to analysis they were thawed at 4 °C. Lipids were extracted using chloroform and methanol (adapted after Folch) and samples were spiked with lipid class-specific internal standards prior to extraction. Samples were resuspended in mass spec acquisition mixture. Samples yielded 16670−29480 pmol of lipids (Dm), 60−238260 pmol of lipids (axolotl plasma and artemia), and 1420−5970 pmol of lipids (axolotl liver). The values obtained are within the optimal range for lipidomic measurements. The lipids samples were analyzed using a shotgun lipidomics platform consisting of the automated sample extraction, an automated direct sample infusion and high-resolution Orbitrap mass spectrometry including lipid class-specific internal standards to assure absolute quantification of lipids. The LipotypeXplorer software was used for the identification of lipids in the mass spectra. Only lipids with an intensity 5x above the noise in the mass spectrum and 5x above the intensity in blank samples were included in the analysis and were considered for final data analysis.

### Data processing of lipidomics results

We only considered lipid species with even fatty acids and those that were present in at least three individuals for our analysis. The amount of excluded total uneven fatty acids was ~1% for flies and 5.50% for Axolotl liver (between 4.37% and 8.47%), on average 10.44% (between 6.63% and 13.05%) Axolotl plasma and on average 11.15% for artemia nauplii. Neutral lipids were normalized relative to the amount of phosphatidylethanolamine (PE).

### Thin Layer Chromatography (TLC)

Thin layer chromatography was performed using various tissue samples, whole blood/hemolymph or fractions after ultracentrifugation. Solid tissue samples were mechanically ground. All samples underwent a Folch-based lipid extraction, followed by vortexing to mix the sample and centrifugation at maximum speed for 5 min. More detailed information about used samples and their dilution prior TLC can be found in Table 1. The samples were air-dried and stored at −20 °C overnight if not used on the same day. Samples were redissolved in chloroform/methanol 2:1 and were pipetted on a 10 cm × 10 cm silica plate (VWR 1056330001). A lipid standard for triacylglycerol, cholesterol and/or cholesteryl ester was applied. Thin layer chromatography was performed with a running buffer separating polar lipids (chloroform/methanol/water in ratios 75/25/2.5) and/or a running buffer separating neutral lipids (heptane/diethyl ether/acetic acid in ratios 70/30/1). The TLC plate was stained with copper sulfate and primulin. The lipid signal was detected using the Amersham slide scanner in the Cy2 channel. Signal intensities were quantified using the Fiji gel analyzer tool^55^.

**Table 1:** Tissues collected for Thin Layer Chromatography (TLC)

Tissue	quantity; volume added	Folch volume and ratio (chloroform/MeOH)	vol. dissolved	vol. on TLC plate
Dm – midgut	2 midguts; 50 µl	200 µl (10:1)	20 µl	3 µl
Dm – fat body	FB cells of 5 flies; 400 µl	650 µl (10:1)	10 µl	4 µl
Dm – hemolymph	5 adult females, 30 µl	200 µl (10:1)	12 µl	10 µl
Axo – plasma	1 µl; 300 µl	250 µl 10:1	20 µl	1 µl
Axo – plasma for gradient TLC	5 µl (1:720 diluted) → 300 µl fractions	1.700 µl 10:1	20 µl	20 µl
Axo – limb/ blastema	1x limb/ blastema; 500 µl	300 µl 10:1	20 µl lower arm 10 µl blastema	1 µl 1 µl
Axo – liver	5 mg; ad 300 mg	250 µl 2:1	40 µl	0.2 µl
Axo – tail, ventral skin, brain, liver, dorsal muscle	3 mg; 90 µl	90 µl 10:1	20 µl	1 µl

### Western blotting

For quantification of the lipoprotein Lipophorin (LPP) in circulating hemolymph of flies, hemolymph of 9 flies got dissolved in 120 µl PBS, followed by a chloroform/methanol protein precipitation. The pellet got resolved in 15 µl 0.1% Triton X-100/PBS. 13 µl sample was mixed with 8 µl 3x SDS-PAGE sample buffer (2% mercaptoethanol). Axolotl liver diluted in 60 µl 0.1% PBS-Triton samples were mechanically homogenized and SDS-PAGE buffer (4x LD and 1% ß-mercaptoethanol) was added. Samples were heat-inactivated at 95 °C and applied on a TRIS-SDS-PAGE (10% separation gel). Separated proteins were transferred on a nitrocellulose membrane using the tank-blotting method. Membranes were blocked for 30 min with 5% BSA in 0.1% Triton X-100/PBS. Primary antibodies were incubated for 2 h at room temperature (Dm) or overnight at 4 °C. The secondary antibodies were incubated at RT for 2 h. The bands were detected using chemiluminescence (Dm: Pierce™ ECL Plus Western Blotting Substrate, Thermo Fisher Scientific, 32132, axolotl: SuperSignal West Pico PLUS (Thermo Fisher Scientific, cat. No. 34580). Used primary antibodies: apo-LPP-II (rabbit, kindly provided by the Eaton Lab^11^, 1:3.000), alpha Tubulin (rabbit, Cell Signaling #2125 1:1.000), anti panAKT (rabbit, #14916 (Thermo Fisher Scientific), 1:1.000), anti p44/42 MAPK (Erk1/2) (137F5) mAb (rabbit, #4695 (Cell Signaling Technologies), 1:1.000), anti-succinate dehydrogenase iron-sulfur subunit (SDHB) (rabbit, Sigma Aldrich, EP288, 1:1000). Used secondary antibodies: rabbit anti HRP (Thermo Fisher Scientific, cat. no. 31466, 1:5.000) for Dm samples, rabbit anti HRP (Cell Signaling Technologies, cat. no. 7074, 1:5.000) for axolotl samples. Original and complete blots are found in Supplementary Fig. 5.

### Western blot quantification

Bands were analyzed using the gel analyzing tool of ImageJ. Background signal was subtracted from band signal intensity. For quantification of the total LPP lipoprotein in circulating hemolymph of flies, signal intensities were normalized to the alpha tubulin signal. For analysis of the density profile of LPP, the band intensities were measured relative to the sum of the signal of all fractions.

### RT-qPCR

Axolotl liver tissue and fly heads were collected and stored in RNA*later*R solution (Thermo Fisher Scientific, cat. no. Am7020) at −20 °C until usage. The liver was homogenized in 500 µl trizol (Thermo Fisher Scientific, cat. no. 15596026), RNA was extracted, purified and eluted in 20 µl H_2_O RNase free. 300 ng RNA was used for cDNA synthesis (Takara, cat. no. RR037A). The cDNA was diluted 1:10 and 4 µl cDNA was added to each RT-qPCR reaction (Promega kit, cat. no. A6001). A standard curve was added to each plate. RPL4 was used as a reference gene and the data was calculated relative to the abundance of intact females.

Primer sequences used for RT-qPCR

AmRPL4 fw: TGAAGAACTTGAGGGTCATGG;

AmRPL4 rev: CTTGGCGTCTGCAGATTTTTT

AmFoxo1 fwd1; GGTGAAGAGTGTGCCCTACT;

AmFoxo1 rev1; GATGCAGCCCTTCTTCTTGG

Primer sequences used for RT-qPCR in Drosophila:

*Dilp2* fwd: ATGGTGTGCGAGGAGTATAATCC;

*Dilp2* rev: TCGGCACCGGGCATG

*Dilp3* fwd: AGAGAACTTTGGACCCCGTGAA;

*Dilp3* rev: TGAACCGAACTATCACTCAACAGTCT

*Dilp5* fwd: GAGGCACCTTGGGCCTATTC;

*Dilp5* rev: CATGTGGTGAGATTCGGAGCTA

### Axolotl gene search and blast

For determining the gene sequences, we performed tblastn searches of the human Apo proteins against the Tanaka axolotl transcriptome (version Am_3.4) and the Whited Lab axolotl transcriptome^18^. To get the protein sequences, we took the part of the axolotl gene hit that matched the human protein sequence and, if needed, extended it up and down the gene hit so that it contained a start and stop codon. The axolotl apolipoprotein sequences were compared with mouse, Xenopus and human protein sequences via NCBI protein blast. The protein size (kD) was determined via a free available online platform^56^.

### Search for conserved axolotl genes

The human CDS of each gene was blasted against the axolotl transcriptome (version Am_3.4). To confirm the identified hit, the axolotl ORF was blasted against human cDNA in Ensembl. For MAPK and AKT, the axolotl protein sequence was phylogenetically clustered using aLeaves^57^. RT-qPCR primers were designed using Primer3 software. The use of human datasets was under the strict accordance with the Declaration of Helsinki.

### Search for sterol metabolism associated genes

Publicly available RNA-seq read data^26^ were downloaded from the axolotl-omics.org website^58^. Adapter sequences and low-quality bases were trimmed from the reads via the programs Cutadapt^59^ and FASTX-Toolkit (Hannon, G.J. (2010) FASTX-Toolkit)^60^, respectively. The reads were then mapped to the most current version of the axolotl genome at the time of writing, AmexG_v6.0-DD^61^, using the software HISAT2^62^. Finally, individual gene expression was quantified with the StringTie software^63^ and differential expression analysis was performed using the edgeR Bioconductor package^64^.

### Statistical analysis

Statistical analysis was performed using GraphPad Prism version 8.1.2 for Windows and Version 9.3.0(345) for MAC and RStudio. All data was tested for normal distribution using the Kolmogorov-Smirnov test. Depending on the outcome, statistical significance was tested using parametric or non-parametric tests. More details on the used tests can be found in the figure legends. Outliers were determined via the ROUT method Q = 1% (Supplementary Fig. 2h). *Drosophila melanogaster* data was always normalized towards the untreated control condition. To account for differences in variance and sample sizes, a Welch’s t-test was performed when indicated in the figure legends. A post hoc power analysis was performed using G*Power: for the mass spec data of axolotl, TAG saturation was used (the most abundant lipid class), and TAG ^C54^ for lipid species measurements and Sterol and SE ratio, all with (α = 0.05, power∼ 0,9).

## Supplementary information

Supplementary Information

Supplementary Data 1

Supplementary Data 2

Supplementary Data 3

Supplementary Data 4

Supplementary Data 5

Supplementary Data 6

Supplementary Movie 1

Supplementary Movie 2

Supplementary Movie 3

Supplementary Movie 4

Supplementary Movie 5

Supplementary Movie 6

## Data Availability

Relevant original mass spectrometry results are provided in Supplementary Data 1, 2, 4 and 5.
