# Supplementary material for: Systemic and local lipid adaptations underlie regeneration in Drosophila melanogaster and Ambystoma mexicanum
Source: NPJ Regen Med. 2024 Oct 29;9:33. doi: 10.1038/s41536-024-00375-x (PMC11522293; doi:10.1038/s41536-024-00375-x)
Supplement: Supplementary file 1 — Supplementary Information [file 41536_2024_375_MOESM1_ESM.pdf]

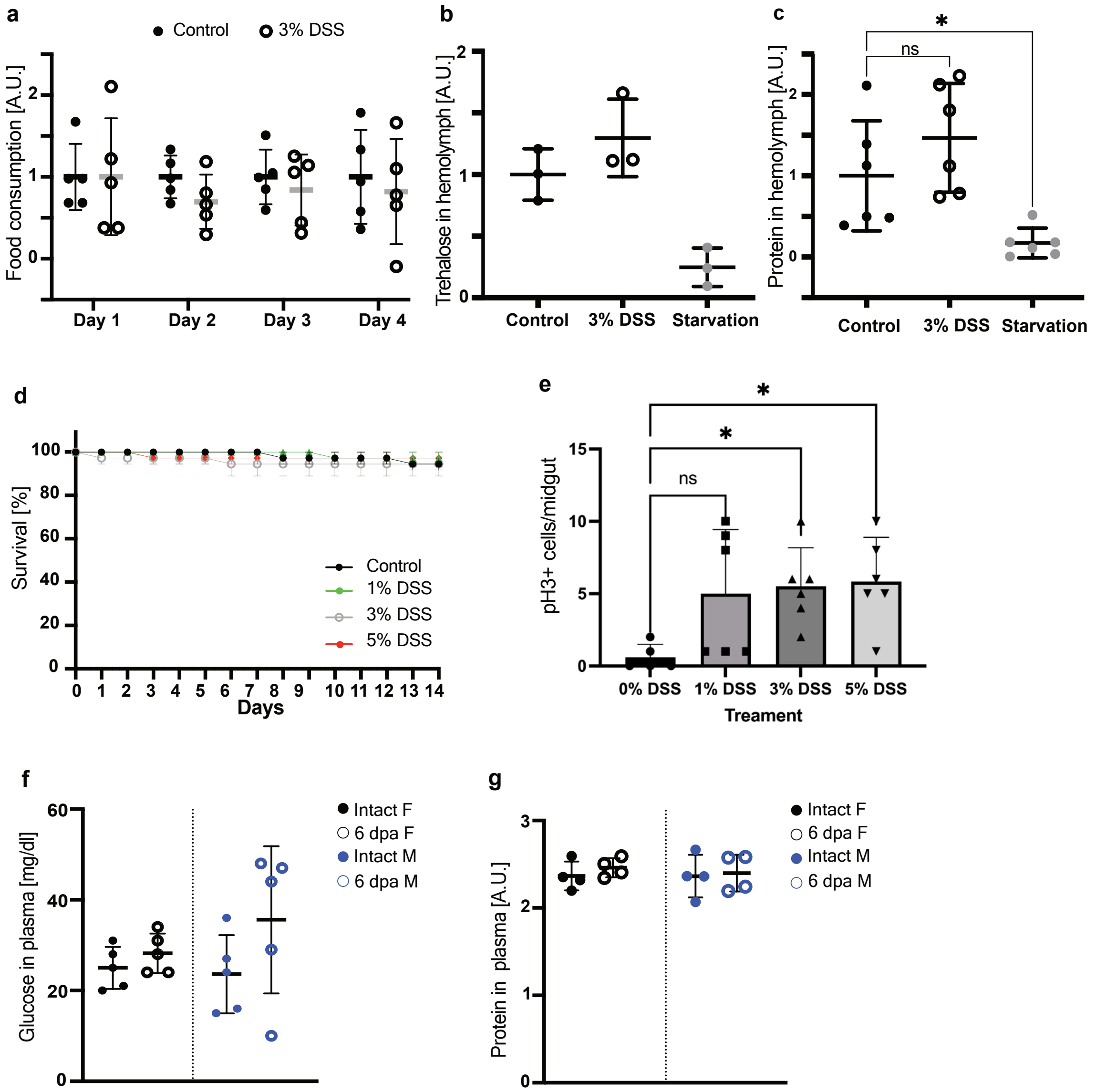
a
Control
3% DSS
Food consumption [A.U.]
0
1
2
Day 1
Day 2
Day 3
Day 4
b
Trehalose in hemolymph [A.U.]
0
1
2
Control
3% DSS
Starvation
c
*
ns
Protein in hemolymph [A.U.]
0
1
2
Control
3% DSS
Starvation
d
Survival [%]
100
80
60
40
20
0
0 1 2 3 4 5 6 7 8 9 10 11 12 13 14
Days
Control
1% DSS
3% DSS
5% DSS
e
*
*
ns
pH3+ cells/midgut
15
10
5
0
0% DSS
1% DSS
3% DSS
5% DSS
Treament
f
Glucose in plasma [mg/dl]
60
40
20
0
Intact F
6 dpa F
Intact M
6 dpa M
g
Protein in plasma [A.U.]
3
2
1
0
Intact F
6 dpa F
Intact M
6 dpa M

Supplementary Figure 1. Feeding behavior, and circulating sugar and proteins in *D. melanogaster* and *A. mexicanum.*

a. Assessment of food consumption in control and regenerating flies using a Capillary Feeder Assay. DSS treatment did not influence the feeding behavior of flies compared to controls (n=60 per condition, 12 flies per vial). Mean and SD are indicated.

b. Analysis of circulating trehalose in circulating hemolymph (blood) of flies. Comparison of control flies on lipid-free food to regenerating flies (3% DSS). n=3 per condition, 27 flies pooled per sample. Mean and SD are indicated.

c. Analysis of circulating protein yields in circulating hemolymph of flies on lipid-free food, during regeneration (3% DSS) and starvation. n=6 per condition, 9 flies pooled per sample. Kruskal-Wallis and Dunn's multiple comparison test, *$p<0.05$. Mean and SD are indicated.

d. Assessment of survival of DSS treated flies on lipid-free food. Treatment with up to 5% DSS at 20°C did not affect the survival of flies compared to controls (n=36 per condition, 12 flies per plate). Mean and SD are indicated.

e. Quantification of intestinal stem cell (ISC) division rates in the whole midgut of adult female fruit flies after 14 day treatment with different concentrations of dextran sodium sulfate (DSS) on lipid-free food. Treatment with 3% DSS was sufficient to induce regeneration in the midgut of flies, shown by an increase of ISC divisions (n=5 for control and n=6 for all other conditions). Mean and SD are indicated.

f, g. Axolotl limb regeneration at 6dpa has no impact on blood glucose and blood protein levels (glucose/protein measurement: n=5/4 per group, N=3). Statistical significance for all graphs was determined by Kruskal-Wallis and Dunn's multiple comparison test. Mean and SD are shown.

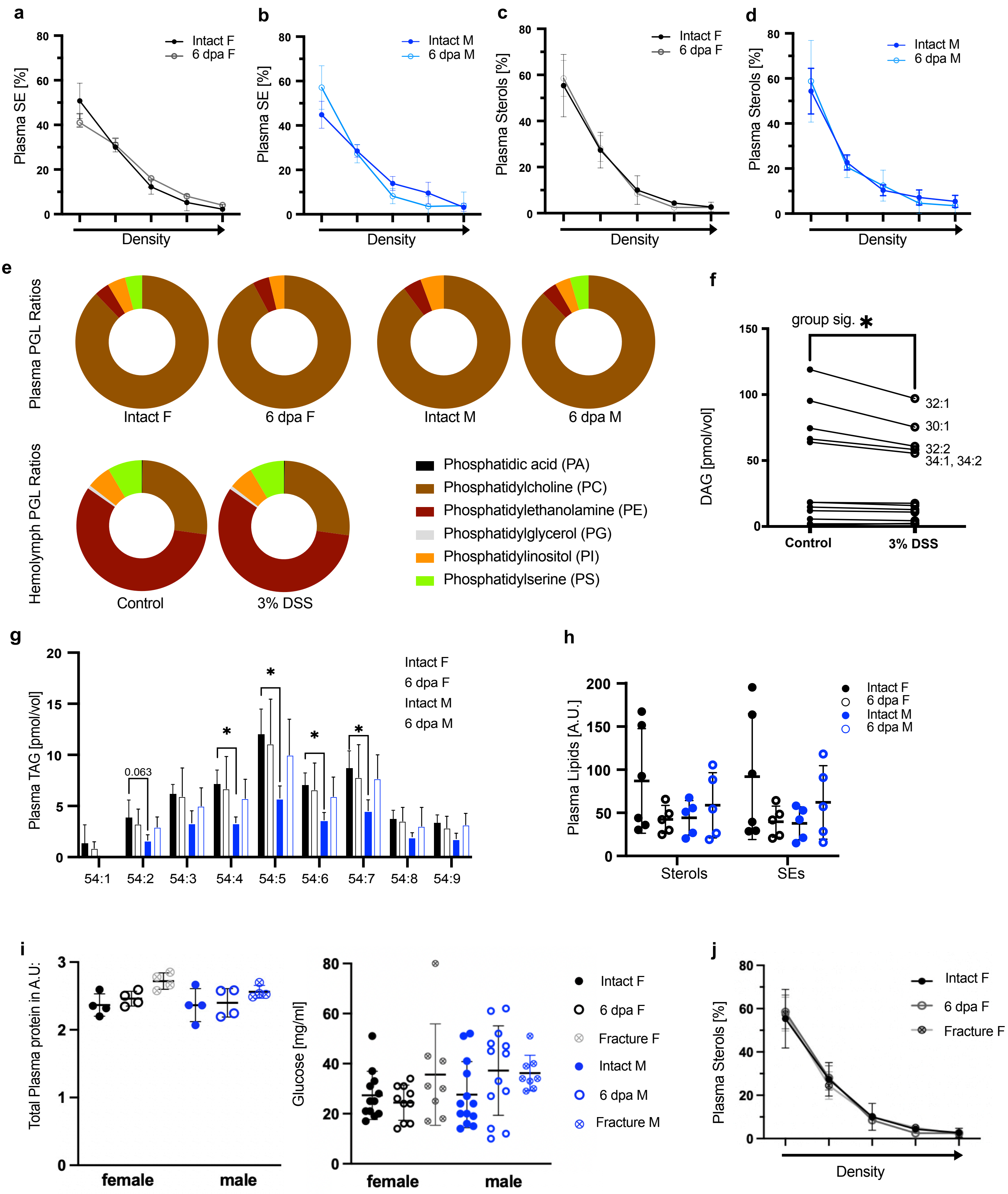

a
Plasma SE [%]
Intact F
6 dpa F
Density
b
Plasma SE [%]
Intact M
6 dpa M
Density
c
Plasma Sterols [%]
Intact F
6 dpa F
Density
d
Plasma Sterols [%]
Intact M
6 dpa M
Density
e
Plasma PGL Ratios
Intact F
6 dpa F
Intact M
6 dpa M
Hemolymph PGL Ratios
Control
3% DSS
Phosphatidic acid (PA)
Phosphatidylcholine (PC)
Phosphatidylethanolamine (PE)
Phosphatidylglycerol (PG)
Phosphatidylinositol (PI)
Phosphatidylserine (PS)
f
group sig. *
DAG [pmol/vol]
32:1
30:1
32:2
34:1, 34:2
Control
3% DSS
g
Plasma TAG [pmol/vol]
Intact F
6 dpa F
Intact M
6 dpa M
0.063
54:1
54:2
54:3
54:4
54:5
54:6
54:7
54:8
54:9
h
Plasma Lipids [A.U.]
Intact F
6 dpa F
Intact M
6 dpa M
Sterols
SEs
i
Total Plasma protein in A.U.
female
male
Glucose [mg/ml]
female
male
Intact F
6 dpa F
Fracture F
Intact M
6 dpa M
Fracture M
j
Plasma Sterols [%]
Intact F
6 dpa F
Fracture F
Density

Supplementary Figure 2. Circulatory adaptations in *D. melanogaster* and *A. mexicanum*.

a-d. Assessment of sex-specific plasma sterol ester (b and c) and sterol (d and e) transport via density gradient TLC in axolotl. The majority of sterol esters and sterols are transported in very low density lipoproteins in both intact and regenerating axolotls of both sex (n=3 per group, N=2). The Mean and SD are indicated.

e. Pie charts of circulatory phospho-glycero lipid (PGL) distribution obtained via mass spectrometry. Phosphatidylcholine (PC) is the most abundant PGL in axolotl plasma and phosphatidylethanolamine (PE) is the most abundant PGL in fly hemolymph. Phosphatidylserine (PS) is present only in intact female and regenerating male axolotl, however not changing in flies. Phosphatidic acid (PA) and phosphatidylglycerol (PG) are only detected as minorities in fly samples. Phosphatidylinositol (PI) levels are similar in both species and do not change due to regeneration. Axolotl data: n=5 per group and fly data: n=6 per group, 3 flies pooled per sample.

f. DAG hemolymph yields in head samples of control and regenerating flies (3% DSS). Identical lipid species are connected using a line to visualize their individual change between the mean of the two experimental groups. Paired t-test of total mean DAG changes, $p^{*}<0.05$. n=6, 3 heads pooled per sample.

g. Quantification of plasma TAG^{C54}, as representative and one of the most abundant circulatory TAG species, in intact and regenerating female and male axolotls via mass spectrometry. Intact males have higher TAG^{C54} levels than intact females. The sex-specific differences are even significant for $TAG^{C54:5}$, which is the most abundant TAG species. During regeneration, females tend to increase their amount of TAG^{C54} in circulation, whereas males maintain rather constant TAG^{C54} levels (n=5 per group). Statistical significance for each lipid group was determined via one way ANOVA and *post hoc* Tukey test. The Mean and SD are indicated.

h. Assessment of axolotl circulatory lipids via TLC. No differences in sterol and sterol ester levels were detected between experimental groups and sex. Statistical significance for each lipid group was determined via one way ANOVA and *post hoc* Tukey test. The Mean and SD are indicated.

i, j. Measurements to compare regenerating animals to animals with an skeletal fracture. i.Total serum protein and glucose measurements in intact, regenerating and fracture groups. Statistical significance for each group was determined via one way ANOVA and *post hoc* Kruskal-Wallis test. j. plasma sterol transport in the same experimental group of Supplementary Figure 2c with the fracture group added (the groups were assessed in the same experiment). For all graphs the mean and SD are shown.

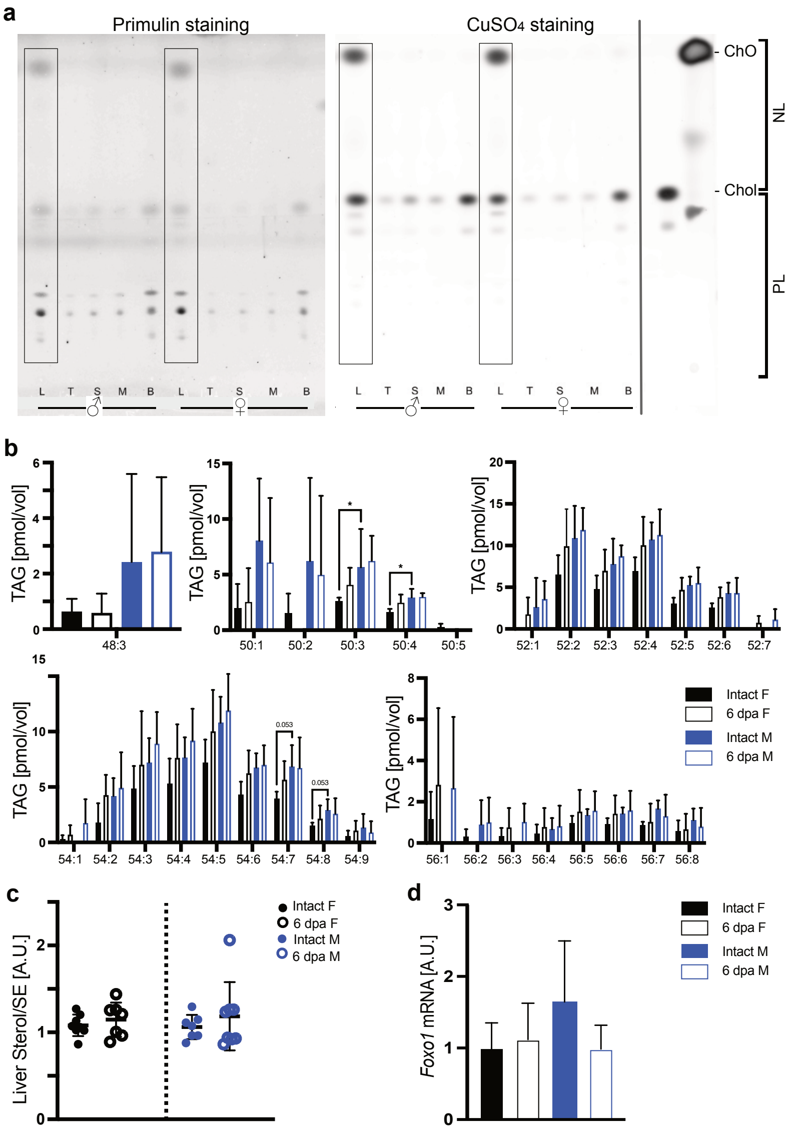
a
Primulin staining
CuSO4 staining
ChO
Chol
NL
PL
L T S M B L T S M B
♂ ♀
b
TAG [pmol/vol]
48:3
50:1 50:2 50:3 50:4 50:5
52:1 52:2 52:3 52:4 52:5 52:6 52:7
54:1 54:2 54:3 54:4 54:5 54:6 54:7 54:8 54:9
0.053
56:1 56:2 56:3 56:4 56:5 56:6 56:7 56:8
Intact F
6 dpa F
Intact M
6 dpa M
c
Liver Sterol/SE [A.U.]
d
Foxo1 mRNA [A.U.]

Supplementary Figure 3. The axolotl liver as lipid storage and its sex-specific changes due to regeneration.

a. Identification of the liver as the predominant lipid storage site in the larval axolotl of males and females via TLC. Tissue applied liver (L), proximal tail (T) right next to the cloaca, belly skin (S), back muscle (M) and brain (B). Primuline staining was performed to detect carbon atoms of polar lipids (PL) and neutral lipids (NL). Copper sulfate staining was used to detect double bonds and carbon rings, thus neutral lipids and sterols (n=1 per sex, 1 technical duplicate).

b. Larval Axolotl Liver Lipid Profile. Quantification of liver lipids in intact and regenerating (6 dpa) axolotls detected via mass spectrometry. TAG species are separated by length (A-E) and the number of DBs per TAG molecule. (A) $TAG^{C48:3}$, (B) TAG^{C50}, (C) TAG^{C52}, (D) TAG^{C54} and (E) TAG^{C56}. All TAGs were normalized to PE levels. Only TAG species that were present in at least 3 individuals per experimental group were considered. n=6. Kruskal-Wallis and Dunn´s multiple comparison test, $*p \leq 0.05$. The mean and SD are shown in all panels. Female (F), male (M).

c. Liver Sterol/SE ratio in intact and regenerating axolotls. n=6 for each female group, n=8 for each male group. One-way ANOVA test and Tukey’s multiple comparison test. Mean and SD are indicated. Female (F), male (M).

d. Quantification of *Foxo1* in the axolotl liver via RT-qPCR (n=5 per group). The expression of *Foxo1* is not significantly changed between experimental groups. Statistical significance was determined via one way ANOVA and *post hoc* Tukey test. The data were normalized relative to intact females. The mean and SD are shown.

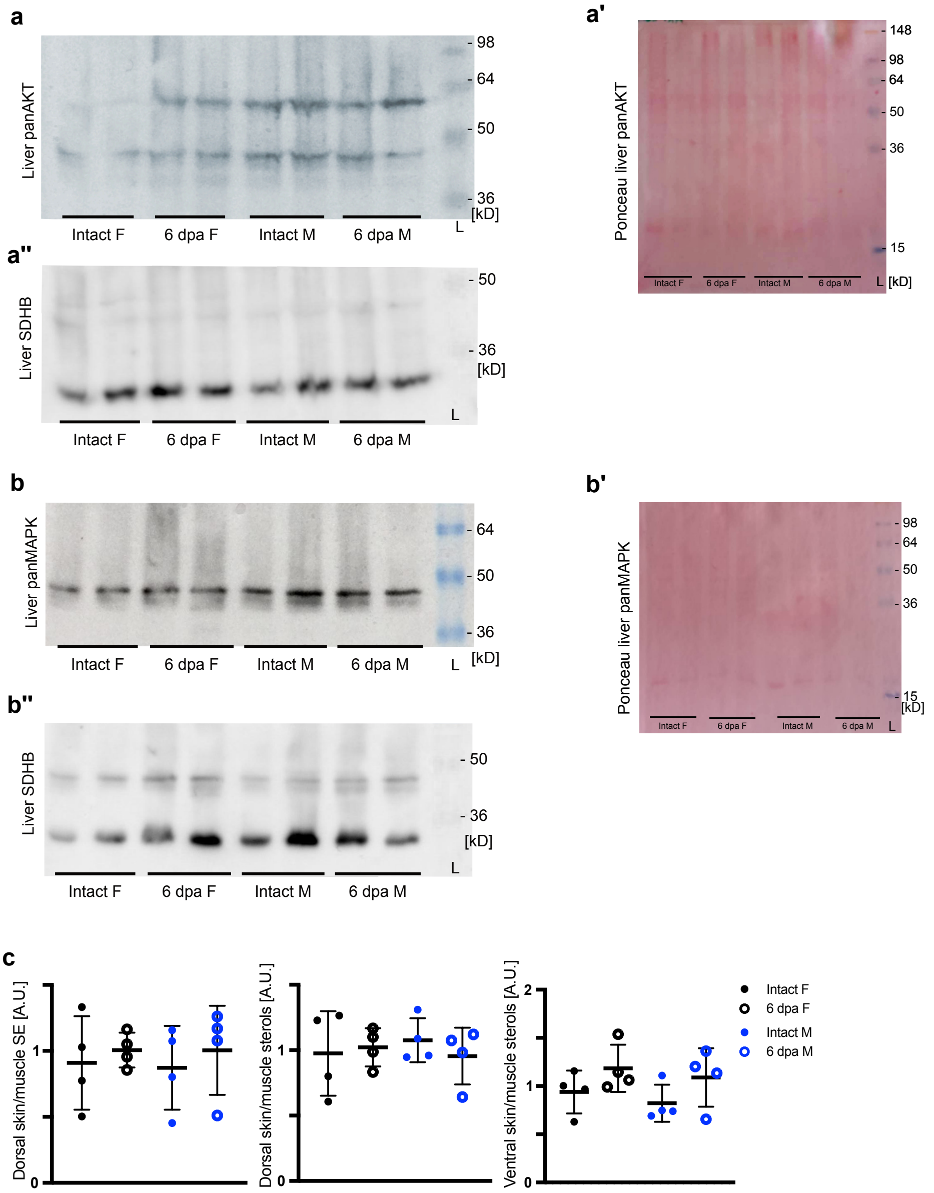

a
Liver panAKT
98
64
50
36
[kD]
L
Intact F
6 dpa F
Intact M
6 dpa M
a'
Ponceau liver panAKT
148
98
64
50
36
15
L [kD]
a''
Liver SDHB
50
36
[kD]
b
Liver panMAPK
64
50
36
[kD]
b'
Ponceau liver panMAPK
98
64
50
36
15
[kD]
b''
Liver SDHB
50
36
[kD]
c
Dorsal skin/muscle SE [A.U.]
Dorsal skin/muscle sterols [A.U.]
Ventral skin/muscle sterols [A.U.]
0
1
2
Intact F
6 dpa F
Intact M
6 dpa M

Supplementary Figure 4. AKT and MAPK protein in axolotl liver, and peripheral tissue sterol content.

a. Axolotl liver AKT protein. Photograph of Western blot loaded with liver samples (n=2) from individual intact or regenerating (6 dpa) female or male axolotls probed for panAKT. Protein standard (L) indicates protein size (kD). In regenerating females, there is a dramatic increase in AKT compared to very low AKT expression in intact females. Male AKT levels do not change due limb regeneration.
a'. Photograph of Ponceau staining matching to (a). 1 mg/ml liver protein was loaded into each lane.
a''. Photograph of the Western blot shown in (a) probed for SDHB as a loading control.
b. Photograph of Western blot loaded with liver samples (n=2) from individual intact or regenerating (6 dpa) female or male axolotls probed for panMAPK. Protein standard (L) indicates protein size (kD). Regeneration has no impact on MAPK levels.
b'. Photograph of Ponceau staining matching to b. 1 mg/ml liver protein was loaded into each lane.
b''. Photograph of the Western blot shown in (b) probed for SDHB as a loading control.
c. Sterol and SE in axolotl dorsal and ventral skin and muscle. Assessment of the Sterol and SE levels in the dorsal skin and muscle and ventral skin and muscle via TLC. Regenerating and intact groups show unchanging Sterol and SE. Ventral skin and muscle had undetectable levels of SE. n=4. Kruskal-Wallis and Dunn's multiple comparison test. Mean and SD are indicated. Female (F), male (M). A.U. arbitrary units.

a

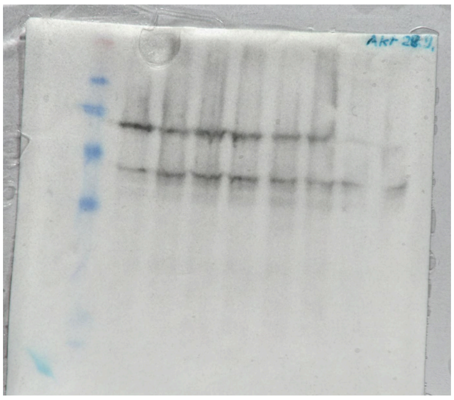

a"

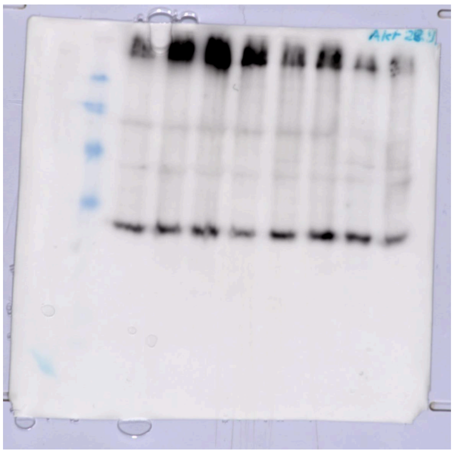

b

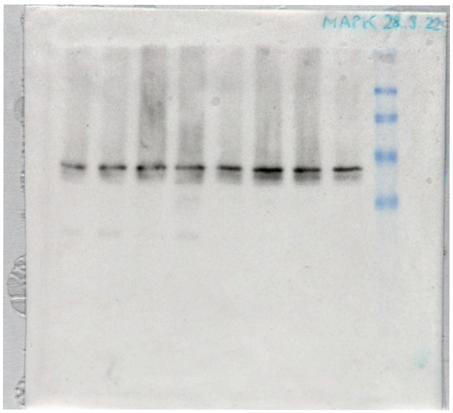

b"

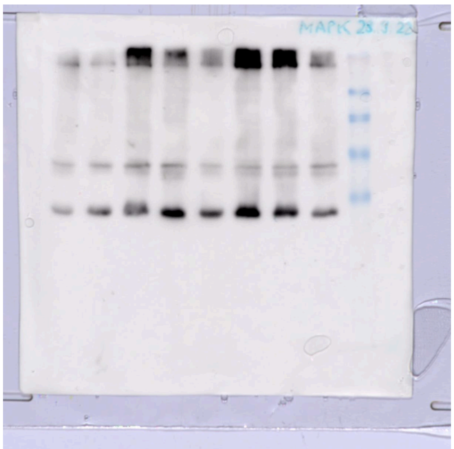

Supplementary Figure 5. Complete and unprocessed western blots for AKT and MAPK and SDHB protein in axolotl liver corresponding to Supplementary Figure 4.

a. Photograph of Western blot loaded with liver samples (n=2) from individual intact or regenerating (6 dpa) female or male axolotls probed for panAKT.

a''. Photograph of the Western blot shown in (a) probed for SDHB as a loading control.

b. Photograph of Western blot loaded with liver samples (n=2) from individual intact or regenerating (6 dpa) female or male axolotls probed for panMAPK.

b''. Photograph of the Western blot shown in (b) probed for SDHB as a loading control.

Supplementary Data 1. Drosophila head mass spec data.

Supplementary Data 2. Artemia Mass spec.

Supplementary Data 3. Axolotl Apolipoprotein gene sequences.

Supplementary Data 4. Axolotl plasma mass spec.

Supplementary Data 5. Axolotl liver mass spec.

Supplementary Data 6. Axolotl AKT and MAPK gene sequences.

Supplementary Movie Legends

Supplementary Movie 1: Drosophila Males fed with fructose
Male flies were video-recorded (1 frame/sec) for 2 hrs. at 20°C.

Supplementary Movie 2: Drosophila Females fed with fructose
Female flies were video-recorded (1 frame/sec) for 2 hrs. at 20°C.

Supplementary Movie 3: Axolotl Females intact
Three-month old axolotl larvae were recorded initially for 15 min (1 frame/sec). Following the initial observation period, 1 ml of artemia-suspension was added and the feeding behavior was recorded for another 2h.

Supplementary Movie 4: Axolotl Females regenerating
Three-month old axolotl larvae were recorded initially for 15 min (1 frame/sec). Following the initial observation period, 1 ml of artemia-suspension was added and the feeding behavior was recorded for another 2h.

Supplementary Movie 5: Axolotl Males intact
Three-month old axolotl larvae were recorded initially for 15 min (1 frame/sec). Following the initial observation period, 1 ml of artemia-suspension was added and the feeding behavior was recorded for another 2h.

Supplementary Movie 6: Axolotl Males regenerating
Three-month old axolotl larvae were recorded initially for 15 min (1 frame/sec). Following the initial observation period, 1 ml of artemia-suspension was added and the feeding behavior was recorded for another 2h.
